# Phenotypic characteristics of aged CD4^+^ CD28^null^ T lymphocytes are determined by changes in the whole‐genome DNA methylation pattern

**DOI:** 10.1111/acel.12552

**Published:** 2016-12-27

**Authors:** Beatriz Suarez‐Álvarez, Ramón M. Rodríguez, Karin Schlangen, Aroa Baragaño Raneros, Leonardo Márquez‐Kisinousky, Agustín F. Fernández, Carmen Díaz‐Corte, Ana M. Aransay, Carlos López‐Larrea

**Affiliations:** ^1^ Department of ImmunologyHospital Universitario Central de AsturiasOviedoSpain; ^2^ Genome Analysis PlatformCIC bioGUNEBizkaia Technological Technology ParkDerioSpain; ^3^ Cancer Epigenetics LaboratoryInstitute of Oncology of Asturias (IUOPA)Hospital Universitario Central de AsturiasOviedoSpain; ^4^ Department of NephrologyHospital Universitario Central de AsturiasOviedoSpain; ^5^ CIBERhed

**Keywords:** aging, CD4^+^CD28^null^ T cells, DNA methylation, gene expression, inflammation, TCR signaling

## Abstract

Aging is associated with a progressive loss of the CD28 costimulatory molecule in CD4^+^ lymphocytes (CD28^null^ T cells), which is accompanied by the acquisition of new biological and functional properties that give rise to an impaired immune response. The regulatory mechanisms that govern the appearance and function of this cell subset during aging and in several associated inflammatory disorders remain controversial. Here, we present the whole‐genome DNA methylation and gene expression profiles of CD28^null^ T cells and its CD28^+^ counterpart. A comparative analysis revealed that 296 genes are differentially methylated between the two cell subsets. A total of 160 genes associated with cytotoxicity (e.g. *GRZB*,*TYROBP,* and *RUNX3)* and cytokine/chemokine signaling (e.g. *CX3CR1*,*CD27*, and *IL‐1R*) are demethylated in CD28^null^ T cells, while 136 *de novo*‐methylated genes matched defects in the TCR signaling pathway (e.g. *ITK*,*TXK*,*CD3G*, and *LCK*). TCR‐landscape analysis confirmed that CD28^null^ T cells have an oligo/monoclonal expansion over the polyclonal background of CD28^+^ T cells, but feature a Vβ family repertoire specific to each individual. We reported that CD28^null^ T cells show a preactivation state characterized by a higher level of expression of inflammasome‐related genes that leads to the release of IL‐1β when activated. Overall, our results demonstrate that CD28^null^ T cells have a unique DNA methylation landscape, which is associated with differences in gene expression, contributing to the functionality of these cells. Understanding these epigenetic regulatory mechanisms could suggest novel therapeutic strategies to prevent the accumulation and activation of these cells during aging.

## Introduction

During differentiation and homeostatic proliferation of CD4^+^ T lymphocytes, some cells lose expression of the CD28 costimulatory molecule, which is fundamental to the activation, proliferation and survival of CD4^+^ T cells (Vallejo *et al*., 2002). CD4^+^ CD28^null^ cells (now known as CD28^null^ T cells) acquire new phenotypic and functional characteristics compared with those of conventional CD4^+^CD28^+^ (named as CD28^+^) T cells (Maly & Schirmer, 2015). An increase in the percentage of CD4^+^ CD28^null^ T cells with age has been reported not only in healthy donors (Weng *et al*., 2009), but also in patients with infections (CMV, HCV, HIV), inflammatory disorders, autoimmune diseases (rheumatoid arthritis, ankylosing spondylitis, multiple sclerosis), and cardiovascular diseases, or in solid organ transplantation (Scarsi *et al*., 2011; Broux *et al*., 2012; Téo *et al*., 2013; Shabir *et al*., 2016). The appearance of this subset during aging can compromise the CD4^+^ T‐cell compartment, leading to a reduced immune response to pathogens and vaccines in the elderly. By contrast, this CD28^null^ subset is only detectable in a low percentage of young and middle‐aged individuals, although it is sometimes highly enriched in the CD4^+^ T‐cell compartment.

The nature of the stimuli that trigger expansion of these CD28^null^ T cells is not fully understood. Some studies suggest that they are generated in response to repeated specific auto‐antigen stimulation, while others show that they respond to ubiquitous antigens such as heat‐shock proteins (Hsp 60) and viral antigens (proteins from CMV), thereby acquiring an innate T‐cell‐like phenotype (Zal *et al*., 2004; van Bergen *et al*., 2009). Some therapeutic approaches have been proposed with the purpose of reducing the percentage of CD28^null^ T cells, such as activation in the presence of IL‐12 or treatments with statins, abatacept, or ATG (Warrington *et al*., 2003; Link *et al*., 2011; Scarsi *et al*., 2011; Duftner *et al*., 2012). However, the basic mechanisms leading to the generation of CD28^null^ T cells remain unknown. Knowledge of them might suggest new therapeutic options to prevent its accumulation and function.

Aging has been associated with gradual DNA methylation changes over the lifespan, marked by global hypomethylation and specific hypermethylation in promoter‐associated CpG islands (Martin, 2005; Heyn *et al*., 2012). Some studies have linked these changes to alterations in the expression levels of DNMT1 and DNMT3B regardless of nutritional habits, lifestyle, or clinical parameters (Ciccarone *et al*., 2016). In this way, age‐associated ‘epigenetic drift’ can potentially restrict the plasticity of T cells while supporting new phenotypes, such as exhausted or senescent T cells (Fraga *et al*., 2005; Issa, 2014). Moreover, it has recently been shown that changes in DNA methylation levels in certain CpG sites are closely associated with the age of individuals (Florath *et al*., 2014). This ‘epigenetic clock’ demonstrates that using a small number of these sites can accurately predict the chronological age of an individual. DNA methylation is a key process in hematopoietic differentiation and the maintenance of subset‐specific T lymphocyte gene expression (Calvanese *et al*., 2012; Rodriguez *et al*., 2015). The regulatory regions of some specific genes and transcription factors are demethylated, and therefore, the genes are overexpressed in certain T subsets (e.g. *IFNG* in Th1 cells), while others are repressed by *de novo* methylation at regulatory regions (e.g. *IFNG* and *FOXP3* in Th2 cells; Suárez‐Álvarez *et al*., 2013). However, these methylation patterns can be altered in response to intrinsic or external stimuli, causing changes in gene expression (Agrawal *et al*., 2010; Suarez‐Alvarez *et al*., 2012). In CD28^null^ T lymphocytes, low levels of Dnmt1 and Dnmt3a lead to a loss of methylation and overexpression of CD70, perforin, and KIR2DL4 (Liu *et al*., 2009).

Here, we examined the whole‐genome DNA methylation and expression profiles of CD28^null^ and CD28^+^ T‐cell subsets. We identified 296 differentially methylated genes related to defects in TCR signaling, cell death pathways, and cytotoxicity ability. Alterations in the expression of genes related to the inflammasome pathway suggest the presence of a preactivated inflammatory phenotype in CD28^null^ T cells. Our results indicate that specific changes in DNA methylation dynamics in conventional CD4^+^ T cells contribute to the acquisition and maintenance of the CD28^null^ phenotype that may predispose individuals to age‐associated decline in their immune response.

## Results

### Overall gain of gene expression in CD28^null^ T cells is associated with changes in immune response and programmed cell death

We analyzed the differential gene expression between CD28^null^ T cells and their CD28^+^ counterparts isolated from healthy donors at different ages to avoid changes due to pathologies. To minimize the differences among individuals, samples were grouped into two pools of 12 donors each (range: 45–75 years; average: 62.1 ± 9.9 years). We identified 1978 differentially expressed genes (DEGs; adjusted *P* < 0.01 and FC ≥ 1.5 or ≤ −1.5), of which 1205 (60.9%) were upregulated and 773 (39.1%) were downregulated in CD28^null^ relative to their CD28^+^ counterparts (Table S1, Supporting information). An overall gain of gene expression was observed in the CD28^null^ T cells regardless of the fold‐change criteria used (Fig. S1A, Supporting information). To study the biological functions involving these genes, we performed GO analysis using more severe criteria (adjusted *P* < 0.01 and FC ≥ 3 or ≤ −3; 545 upregulated and 125 downregulated genes). We observed that GO terms related to immune response (GO: 0006955), regulation of programmed cell death (GO: 0043067), defense response (GO: 0006952), and cell activation (GO: 0001775) were markedly enriched in CD28^null^ T cells (Fig. S1B and Table S2, Supporting information). Genes related to immune and defense responses were upregulated in CD28^null^ cells, while loss of expression was associated with a wider range of functions, such as response to stress, the macromolecule catabolic process, chromatin modifications, and cell death (Fig. S2 and Table S2, Supporting information). KEGG analysis showed that DEGs are enriched in pathways related to autoimmune diseases, infections or allograft rejection, in which the functionality of these CD28^null^ T cells had been previously demonstrated (Fig. S1C and Table S2, Supporting information).

**Figure 1:**
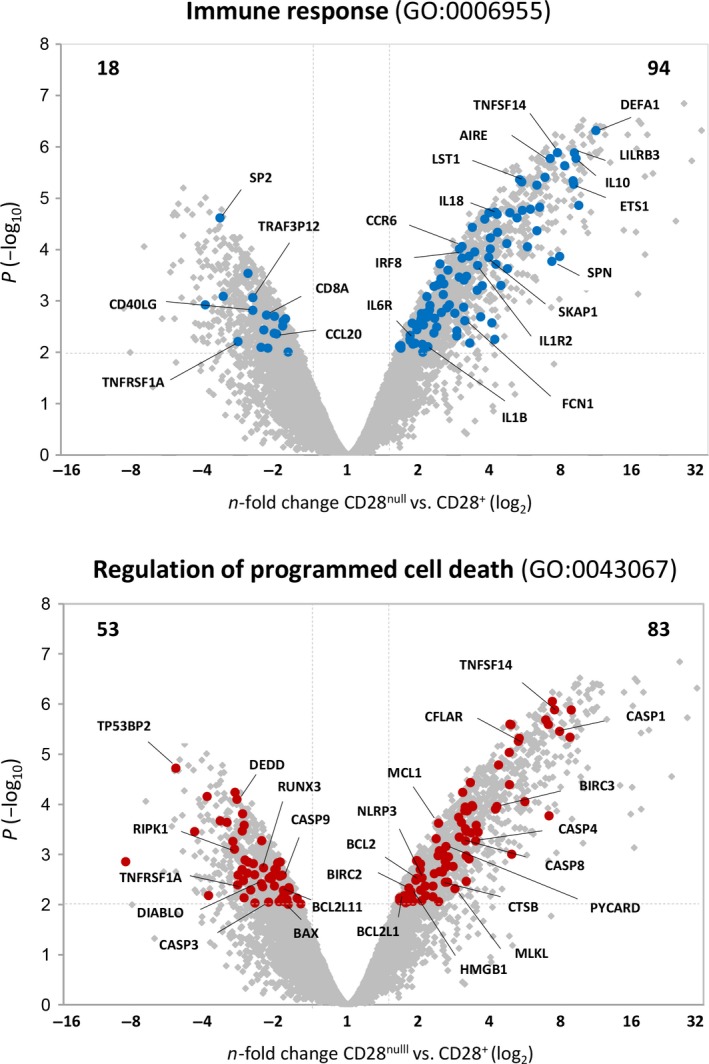
Volcano plots of genes associated with the most representative categories, immune response and regulation of programmed cell death, and differentially expressed in CD28^null^ T cells. Gray dots indicate all DEGs between CD28^+^ and CD28^null^ T cells. Genes associated with the immune response (GO:0006955) and programmed cell death (GO: 0043067) categories are highlighted in blue and red, respectively. For this analysis, the criteria of adjusted *P* < 0.01 and ≥ 1.5‐fold change in size were used. Numbers in the corners show the number of genes upregulated and downregulated in CD28^null^ T cells in each functional category.

We then analyzed in detail the two most significantly altered functional categories—immune response and regulation of programmed cell death—by considering all the DEGs within each category (*P* < 0.01 and FC ≥ 1.5 or ≤ −1.5). Our results showed that most of the DEGs related to the immune system (94 of 112 genes, 84%) were overexpressed in CD28^null^ T cells, suggesting a gain of immune functions in these cells (Fig. 1). Some of these genes correspond to receptors that are strongly expressed in NK cells (*LILRB3*,* LILRB2*), chemokines (*CCR6*,* CCL20*), cytokines (*IL‐10*,* IL‐18*,* IL‐6R*,* IL‐1R2*), and adhesion molecules (*CD14*,* CD1C*,* CD86*,* TNFSF8*,* TNFSF13B*,* TNFSF14*), which regulate the migration and recruitment of other cell types. The genes involved in the interferon (IFN) pathway, such as IRF8 transcription factor (TF), were also more strongly expressed. This gene regulates expression of LST1 protein, which inhibits lymphocyte proliferation. Other autoimmunity‐related TFs, such as ETS‐1 and AIRE, were also upregulated. Conversely, the loss of *CD40LG* gene expression in CD28^null^ T cells could involve defects in the interaction and activation of B cells.

One of the main characteristics of CD28^null^ cells is their resistance to programmed cell death (Kovalcsik *et al*., 2015). We observed a clear deregulation of the expression levels of the Bcl‐2 (B‐cell lymphoma‐2) family members. CD28^null^ T cells expressed high levels of the antiapoptotic genes *BCL2*,* BCL2L1* (encoding Bcl‐x), and *MCL‐1*, while the pro‐apoptotic genes *BAX* and *BCL2L11* (*BIM*) were significantly downregulated (Fig. 1). These data correlates with the low levels of Annexin V staining observed in CD28^null^ T cells before and after staurosporine‐induced apoptosis and in comparison with their CD28^+^ counterparts (Fig. S3, Supporting information). Moreover, expression of negative apoptosis regulators such as *CFLAR* (also known as *FLIP* or *CASPER*), *BIRC2* (cIAP‐1) and *BIRC3* (cIAP‐2) was stronger in these cells, in contrast to the downregulation of the positive regulator *DIABLO*, which is involved in caspase activation. Other genes related to necroptosis, such as *RIPK1* and *MLKL*, were also underexpressed in CD28^null^ cells.

### CD28^null^ T cells overexpress genes involved in the inflammasome pathway

**Figure 2:**
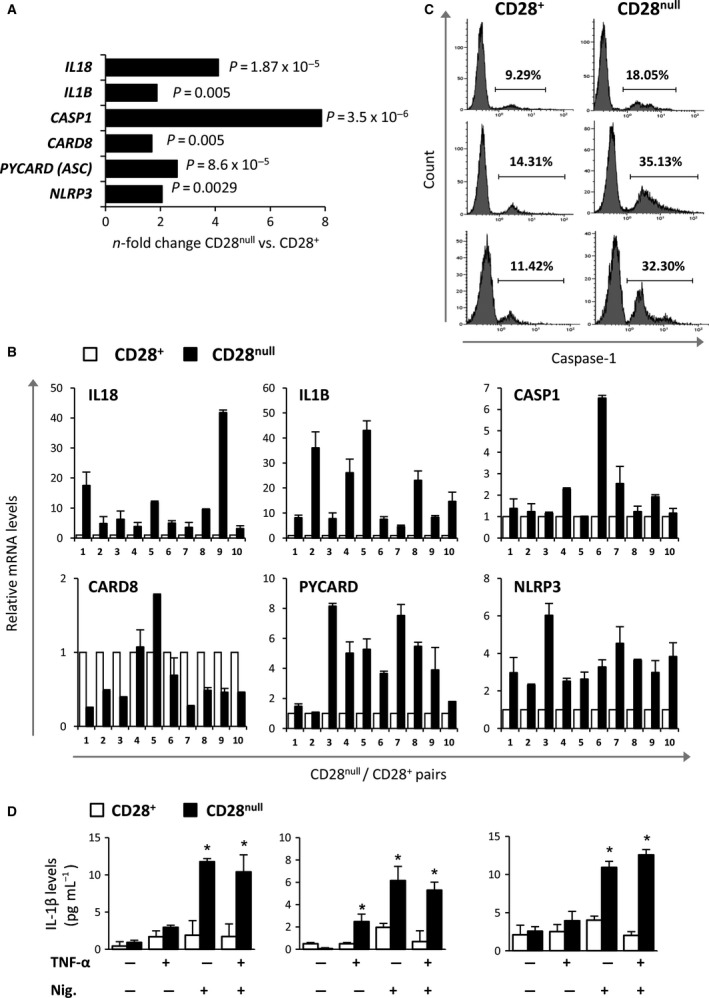
Overexpression of the inflammasome pathway in CD28^null^ T cells. (A) Difference in the expression of genes related to the inflammasome in CD28^null^ T cells compared with CD28^+^ T cells based on whole‐genome expression array data. (B) RT–PCR analysis of inflammasome genes (pro‐*IL‐1B*, pro‐*IL‐18*,*CARD8*,*CASP‐1*,*NLRP3,* and *PYCARD*) in CD28^+^ and CD28^null^ T‐cell subsets isolated individually from 10 healthy donors. (C) Histograms showing the expression level of the active form of caspase‐1 at baseline in CD28^+^ and CD28^null^ T cells isolated from three healthy donors. For this purpose, the FLICA probe, which binds only to the active form of caspase, was added to the cell culture and further detected by flow cytometry. (D) IL‐1β levels quantified by ELISA in the cell supernatants of CD28^null^ and CD28^+^ T cells before and after overnight stimulation with TNF‐α (5 ng mL^−1^), nigericin (10 μm), or both. Histograms are representative of three independent experiments and assayed by duplicates. * P <0.05 versus CD28^+^ T cells.

We found that all genes comprising the NLRP3‐inflammasome complex were overexpressed in CD28^null^ cells (Fig. 2A). These included the NOD‐like receptor (NLR) pyrin domain containing 3 (*NLRP3*), the ASC adaptor protein encoded by the *PYCARD* gene, the caspase recruitment domain family member 8 (*CARD8*), and the inactive pro‐form caspase‐1 (*CASP1*). Together, they form a multimolecular protein complex, which is essential for the cleavage of caspase‐1 into its active form. The pro‐forms of the *IL‐1B* and *IL‐18* genes were also significantly more strongly expressed in CD28^null^ T cells. To corroborate these findings, we analyzed the expression of these genes in paired CD28^null^/CD28^+^ T‐cell samples isolated individually from 10 healthy donors. We confirmed that the expression levels of *NLRP3*,* PYCARD* (*ASC*), *IL‐1B,* and *IL‐18* were higher in all CD28^null^ T‐cell samples (Fig. 2B). Nonetheless, the inactive form of caspase‐1 was only more strongly expressed in four of ten (40%) samples, and the *CARD8* gene expression levels were often downregulated in CD28^null^ T cells. We also observed that in the baseline state, CD28^null^ T cells showed higher active caspase‐1 levels than their CD28^+^ counterparts (Fig. 2C), and under nigericin stimulation, they were able to release an active form of the pro‐inflammatory IL‐1β cytokine (Fig. 2D). Nigericin alone, but not TNF‐α, was sufficient to activate caspase 1 and induce the release of IL‐1β in CD28^null^ T cells, suggesting a basal preactivating state in these cells.

### Changes in the DNA methylation profile in CD28^null^ T cells contribute to altered TCR signaling and cytotoxicity ability

**Figure 3:**
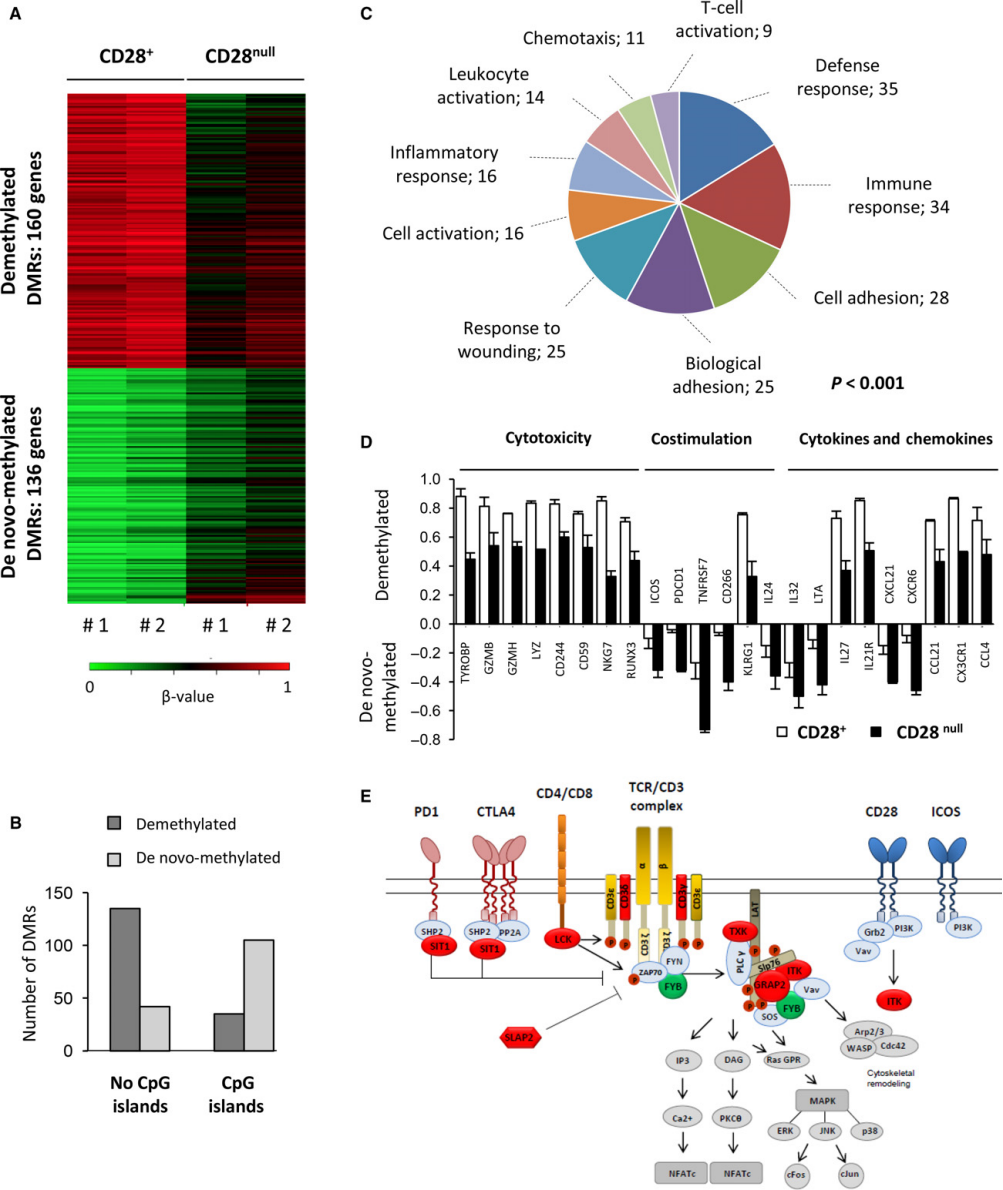
Overview of the differentially methylated regions in CD28^null^ T cells and their functional implication. (A) Heat map showing regions differentially methylated (DMRs) between CD28^+^ and CD28^null^ T cells. A set of 317 probes (296 genes) differed between the T‐cell subsets. Datasets were generated from two biological replicates per cell type (#1 and #2), obtained from a pool of 12 healthy donors each, to minimize the differences among individuals. Samples were pooled using the same DNA quantity per donor. The methylation levels vary from unmethylated (β = 0, red) to fully methylated (β = 1, green). (B) Distribution of DMRs in CD28^null^ T cells according to localization in a CpG island or not. (C) Gene ontology (GO) analysis of all DMRs in CD28^null^ T cells (160 genes demethylated and 136 genes *de novo*‐methylated) with adjusted *P* < 0.01. The ten most significant categories and the number of genes in each are shown. (D) Genes associated with the immune response (cytotoxic molecules, costimulatory molecules, cytokines, and chemokines) and differentially methylated in CD28^null^ T cells. (E) Illustration of the altered T‐cell receptor (TCR) signaling pathway in CD28^null^ T cells. *De novo*‐methylated genes are highlighted in red; demethylated genes are shown in green.

We examined whether changes in gene expression of CD28^null^ T cells were due to alterations in the whole‐genome methylation profile, using DNA isolated from the same pools as before. Unsupervised clustering analysis and scatterplots revealed the reproducibility of the two pools in both cell types (Fig. S4, Supporting information). We detected 317 probes or DMRs between CD28^null^ and CD28^+^ T‐cell subsets (Table S3, Supporting information). Of these, 170 probes (160 genes) corresponded to demethylated regions in CD28^null^ T cells, and 147 probes (136 genes) were *de novo*‐methylated regions (Fig. 3A). Most regions that lost DNA methylation were located outside CpG islands, while gain of methylation was associated with CpG islands (Fig. 3B).

To determine the functional relevance of these changes, we performed GO analysis (Fig. 3C), which revealed an enrichment of cellular functions related to the functionality of CD4^+^ T lymphocytes, such as defense response, immune response, cell adhesion, response to wounding, cell activation, and inflammatory response. A detailed analysis of these categories is shown in Fig. S5 and Table S4 (Supporting information). Differentially methylated genes were associated with costimulatory molecules (*ICOS*,* PDCD1*,* CD27*,* CD226*), cytokines (*IL‐19*,* IL‐27*,* IL‐24*,* IL‐32*,* IL‐21R*,* LTA*), chemokines (*CCL21*,* CX3CR1*,* CXCL1*,* CCL4*,* CXCR6*), adhesion molecules (*ITGAL*,* KLRG1*,* LY9*), apoptosis‐related molecules (*BCL2*,* FASLG*), T‐cell activation (*WAS*,* LCK*,* SLA‐*2), and cytotoxicity ability (*TYROBP*,* GZMB*,* GZMH*,* LYZ*,* CD244*,* CD59*,* NKG7*,* RUNX3*; Fig. 3D). KEGG analysis revealed that *de novo*‐methylated genes in CD28^null^ T cells were mainly associated with the T‐cell receptor (TCR) signaling pathway, whereas demethylated genes were mainly involved in NK‐mediated cell cytotoxicity and cytokine/chemokine signaling (Table 1).

**Table 1:** KEGG pathway of differentially methylated genes in CD28^null^ T cells

	Pathway	*P*	Genes
*De novo*‐methylated genes			
hsa04660	T‐cell receptor signaling pathway	2.25E‐04	*ITK, CD3G, CD3D, ICOS, LCK, GRAP2, PDCD1*
hsa04060	Cytokine–cytokine receptor interaction	0.0194	*CXCL1, CXCR6, FASLG, IL‐24, BMPR1B, LTA, EPO*
hsa05340	Primary immunodeficiency	0.0333	*CD3D, ICOS, LCK*
Demethylated genes			
hsa04062	Chemokine signaling pathway	0.0016	*FGR, CCL21, NCF1, PREX1, CX3CR1, CCL8, WAS, CCL4*
hsa04060	Cytokine–cytokine receptor interaction	0.0027	*LTBR, CCL21, IL‐19, IL‐21R, CX3CR1, CCL8, CSF3R, IFNGR2, CCL4*
hsa04650	Natural killer cell‐mediated cytotoxicity	0.0358	*CD244, GZMB, FCGR3B, IFNGR2, TYROBP*

**Figure 4:**
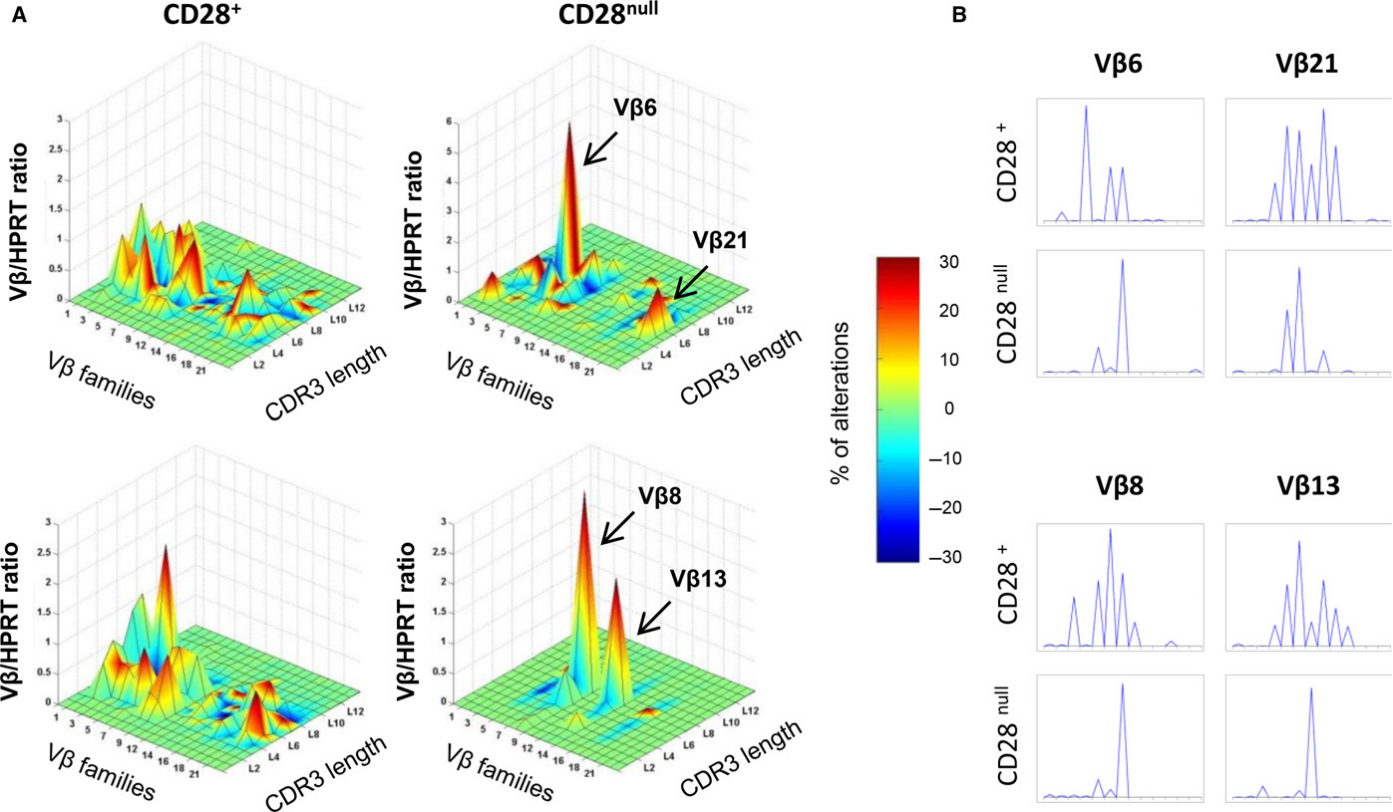
Alterations of the TCRVB repertoire between CD28^+^ and CD28^null^ T cells. (A) Quantitative and qualitative analysis of TCR repertoire was performed in CD28^+^ and CD28^null^ T cells isolated from two healthy donors. Results are displayed as a three‐dimensional TcLandscape^®^ in which the X, Y, and Z axes represent the 26 human Vβ families, the Vβ/HPRT (nonregulated gene) ratios, and the CDR3 lengths, respectively. The percentages of CDR3‐LD alterations are represented as a color code from deep blue (−30%) to dark red (+30%). (B) Histograms showing the spectratype analysis of CDR3 lengths for Vβ families with the highest Vβ/HPRT ratio in the CD28^null^ T‐cell subset for each donor.

Senescent CD28^null^ T cells are not able to mount a robust proliferative response to stimulation (Chou & Effros, 2013). We found many genes associated with TCR activation and signaling to be *de novo*‐methylated in CD28^null^ cells (Fig. 3E), for example, the CD3 complex (*CD3G* and *CD3D*), protein tyrosine kinase (PTK) genes (*LCK*,* ITK*,* TXK*), the Grb‐2‐related adaptor protein 2 (*GRAP2*), and the adapter proteins *SIT1* (signaling threshold regulating transmembrane adaptor 1) and *SLA2* (Scr‐like adaptor 2), which negatively regulate TCR signaling. Only the gene encoding the FYN‐binding protein, *FYB*, was demethylated in CD28^null^ T cells. Additionally, we examined T‐cell diversity and selection by analyzing TCR (β‐chain) usage biases between the CD28^+^ and CD28^null^ T‐cell subsets (Fig. 4A). The Vβ transcription profile revealed higher Vβ/HPRT ratios in CD28^null^ T cells, corresponding to specific families in each sample (Vβ6 and Vβ21 in the first individual; Vβ8 and Vβ13 in the second), which accumulate a high percentage of alterations, as shown by the dark red color. The length distribution of CDR3 in CD28^+^ T cells shows a Gaussian profile (polyclonal), while CD28^null^ T cells show a specific CDR3 length distribution, resembling an oligo/monoclonal expansion (Fig. 4B).

### CpG methylation is inversely correlated with gene expression in CD28^null^ T cells

Changes in DNA methylation are directly related to modifications in chromatin structure and gene expression. We analyzed the association between differentially methylated and expressed genes in CD28^+^ and CD28^null^ T cells based on array data. We took data from 170 genes, of which 31 (18.23%; *LILRB3*,* CX3CR1*,* GZMB*,* BCL2,* or *TYROBP* among others) were demethylated and had a higher level of expression in CD28^null^ T cells, and 13 (7.64%) genes (such as *ITK*,* LY9*,* SLA2,* or *LTA*) were *de novo*‐methylated and downregulated in CD28^null^ T lymphocytes (Fig. S6, Supporting information). Additionally, we corroborate that CD28^null^ T cells show a lesser expression of Lck that leads to a diminished phosphorylation of ZAP70 (Fig. S7, Supporting information). All other differentially methylated genes without changes in gene expression may be primed for further alteration upon T‐cell activation.

**Figure 5:**
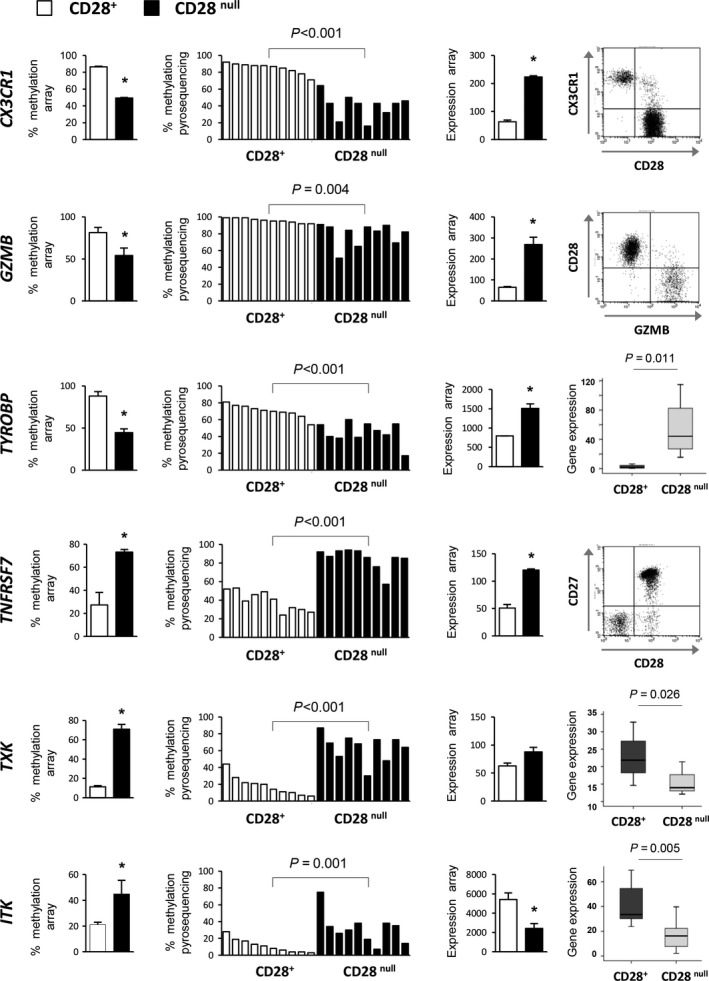
Validation of differentially methylated and expressed genes in CD28^+^ and CD28^null^ T‐cell subsets isolated individually from ten healthy donors. For each gene is shown (from left to right): percentage of methylation based on data from the Illumina Infinium HumanMethylation27 BeadChip array; methylation level determined by bisulfite pyrosequencing analysis in CD28^+^ and CD28^null^ T‐cell subsets isolated individually from ten healthy donors; mRNA expression levels based on the Illumina expression array; gene expression analyzed by flow cytometry or qRT–PCR in CD28^+^; and CD28^null^ T‐cell subsets isolated individually from ten healthy donors. Dot plots are representative of one of the ten experiments. Box plots show the average expression levels of ten donors.* P < 0.05 versus CD28^+^ T cells.

We selected six genes on the basis of their immunological relevance to validate and replicate the array‐based results. Methylation was analyzed by bisulfite pyrosequencing, and expression levels were evaluated by flow cytometry or qRT–PCR in CD28^+^ and CD28^null^ T‐cell subsets isolated from 10 healthy donors (range: 48–92 years; average 70.2 ± 11.6 years). There was a strong correlation between the methylation level and expression status (Fig. 5), for example, the chemokine *CX3CR1*, which is involved in cellular migration, was demethylated and highly expressed on the cell surface of CD28^null^ T cells. Similar results were obtained for the adapter molecule TYROBP (also known as *DAP12*) and the cytotoxic molecule GZMB. Both molecules had a higher level of expression in CD28^null^ T cells due to demethylation, although the degree of demethylation in *GZMB* was dependent on each donor. Demethylation of the *DAP12* locus could facilitate its expression in CD28^null^ cells, thereby acting as an activating signal transduction element and enhancing its cytotoxicity ability. Conversely, the costimulatory molecule *TNFRSF7* (*CD27)* was highly methylated in CD28^null^ cells. Although the gene expression array did not confirm the loss of CD27 expression in CD28^null^ T cells compared with their CD28^+^ counterparts, flow cytometry corroborated that all CD28^null^ T cells lacked this costimulatory molecule. The PTK genes, *ITK* and *TXK*, were *de novo*‐methylated in CD28^null^ T cells, causing their expression to be downregulated.

## Discussion

One of the most consistent age‐associated changes in human T cells that contribute to a decline in immune function is the accumulation of CD28^null^ T cells. A high frequency of CD28^null^ T cells (up to 50% of total CD4^+^ T lymphocytes) has been described in aging and several pathologies associated with chronic inflammatory processes, but also in some healthy young and middle‐aged individuals (Weng *et al*., 2009). The generation of this subset has been mostly attributed to pro‐inflammatory environments and repeated antigen stimulation, but the molecular mechanisms that contribute to its accumulation and maintenance remain to be elucidated. To date, most studies have been carried out with CD28^null^ T cells isolated from patients whose disease etiology, treatments, and concomitant effects differed substantially, increasing the variability associated with this cell subset. Our study, carried out with healthy donors at different ages, demonstrates that DNA methylation contribute to the functionality and characteristics of CD28^null^ T lymphocytes. This is the first report of whole‐genome DNA methylation and expression profiles of CD28^null^ and its CD28^+^ counterpart. Changes in DNA methylation levels in the promoter regions of specific genes related to the immune response, TCR signaling, and cell death pathways reveal epigenetic control during differentiation to CD28^null^ T cells.

DNA methylation is an essential regulatory mechanism of key processes of T lymphocytes, such as differentiation to effector cells or the establishment of a unique phenotype in the memory cells (Li *et al*., 2009; Suarez‐Alvarez *et al*., 2012; Komori *et al*., 2015). Previous studies have observed global DNA demethylation with aging associated with a lower level of DNMT1 and DNMT3A/B expression. There is a linear decrease in DNMT3B expression with age, while the levels of DNMT1 only gradually decreased up to the age of 64 years (Ciccarone *et al*., 2016). Moreover, Li *et al*. (2009) reported that in ‘senescent’ CD28^null^ T cells, decreased DNMT1 and DNMT3A expression causes demethylation and overexpression of *CD70*,* PRF,* and *KIR2DL4* genes. Here, we show that changes in the global DNA methylation profile in CD28^null^ T cells are equally associated with gain and loss of methylation in specific genes. We observed that demethylated regions are mainly associated with genes related to immune function, such as cytotoxicity response, cytokine/chemokine signaling, or costimulation. Results confirmed by bisulfite sequencing, the gold standard for methylation studies, show that CD28^+^ T cells isolated from healthy donors of various ages have methylation levels > 80% for *CX3CR1*, while their CD28^null^ counterparts show levels of 20–40%. When we examined the expression of this chemokine by expression arrays and flow cytometry, all CD28^null^ T cells showed a higher level of expression of CX3CR1, but the CD28^+^ subsets lacked this receptor. CX3CR1, which is expressed mainly in NK, CD8^+^ T, dendritic, endothelial and epithelial cells, and monocytes, but not in CD4^+^ T lymphocytes, is involved in T‐cell migration to inflamed tissue in response to a gradient of their ligand, fractalkine (CX3CL1; Fong *et al*., 1998). Thus, DNA demethylation in the *CX3CR1* gene promoter confers on the CD28^null^ T cells the ability to migrate toward inflammatory tissues, where they exert their inflammatory and cytotoxic effects. Overexpression of CX3CR1 in IL‐7Rα^low^ effector‐memory CD8^+^ T cells is also known to be due to loss of methylation (Shin *et al*., 2015). We observed that expression of most genes associated with the cytotoxic ability acquired by CD28^null^ T cells is also regulated by DNA methylation. These include the following: TYRO protein tyrosine kinase binding protein (*TYROBP*), which acts as an activating signal transduction element associated with KIR family proteins (Mulrooney *et al*., 2013); CD59 cell surface glycoprotein, which functions as a coreceptor and activates human NK‐cell‐mediated cytotoxicity (Marcenaro *et al*., 2003); NKG7 (GMP‐17) granule membrane protein (Medley *et al*., 1996); RUNX3 transcription factor, which regulates the expression of genes involved in the differentiation of CTLs such as *EOMES*,* IFNG*,* PRF1* (Cruz‐Guilloty *et al*., 2009), and the cytotoxic molecules, granzyme B and H. Our results demonstrate the essential role of DNA demethylation in conferring cytotoxic properties on differentiated CD28^null^ T cells.

The costimulatory molecule CD28 plays multiple roles in T‐cell activation, proliferation, and survival. These CD4^+^ T lymphocytes that lose CD28 expression display striking features, such as reduced T‐cell receptor (TCR) diversity, and defects in the antigen‐induced proliferation compromising the immune response (Maly & Schirmer, 2015). Quantitative analysis of the TCR repertoire demonstrates that CD28^null^ T cells are highly enriched in specific Vβ families and show an oligo/monoclonal expansion of the CDR3 segments, leading to a constrained immune response that varies between individuals. This suggests that CD28^null^ T cells might be generated in response to repeated antigen exposure, although antigens differ between individuals and pathologies. We also found that genes associated with TCR signaling are *de novo*‐methylated in CD28^null^ T cells. Following TCR engagement, Lck is brought into proximity of the CD3 complex and phosphorylates ITAM motifs to further recruit the Zap70 protein (Huse, 2009). After that, Zap70 can be phosphorylated by Lck and together with the Itk and Txk kinases phosphorylate proteins of the LAT/Slp76 complex. These sequential steps are essential to lead to T‐cell activation and proliferation. In CD28^null^ T cells, we show that hypermethylation of *LCK* gene is associated with less phosphorylation of the Zap70 that could damage TCR‐mediated initial activation (Limbach *et al*., 2016). Moreover, *de novo* methylation of *ITK* and *TXK* genes reduces the expression of these kinases. A decline in levels of tyrosine‐phosphorylated proteins with age had been associated with defects in TCR activation pathways (Pawelec *et al*., 2001). The decrease in Lck in CD28^null^ T cells may result in partial TCR activation and trigger proliferative hyporesponsiveness. The greater overexpression of the KLRG1 co‐inhibitory receptor in CD28^null^ T cells by loss of methylation could interfere with T‐cell proliferation via Akt‐mediated changes in cyclins and cyclin inhibitors (Henson *et al*., 2009). Thus, epigenetic silencing of key genes of the TCR pathway may contribute to the slower division of CD28^null^ T cells in response to antigen activation.

By contrast, we observed that genes involved in the inflammasome configuration (*NLRP3*,* CASP‐*1, *PYCARD*,* IL‐1B,* and *IL‐18*) and caspase‐1 activation (*HMGB1* and *CTSB)* are stronger upregulated in CD28^null^ T cells vs. their CD28^+^ counterparts. These expression changes lead to a higher susceptibility of these cells to the activation of the inflammasome and IL‐1β release. Thus, CD28^null^ T cells could show a baseline preactivation state that under certain environmental stimuli contributes to trigger a chronic inflammatory process as had been reported in CD4^+^ T cells from HIV‐1 patients (Doitsh *et al*., 2014).

The longevity and persistence over time of CD28^null^ T cells has been associated with a high apoptosis resistance (Vallejo *et al*., 2000). Previous studies and data from our expression arrays indicate that genes involved in the extrinsic and intrinsic apoptosis pathways are differentially expressed in CD28^+^ and CD28^null^ T cells. In CD28^null^ T cells, the FasL death receptor‐dependent pathway is mainly impaired by increased expression of the antiapoptotic molecule FLIP (also known as *CASPER* or *CFLAR*), which interacts with caspase‐8 due to its structural similarity (Majkut *et al*., 2014). Alterations in the mitochondrial‐dependent pathway are attributed to the lower expression levels of the Bim and Bax pro‐apoptotic molecules in these cells. Unlike what was noted by Kovalcsik *et al*. (2015), we observed significant overexpression of the antiapoptotic molecules Bcl‐2, Bcl‐x (*BCL2L1* gene), and Mcl‐1 (*BCL2L3* gene). The *BCL2* gene is significantly demethylated in CD28^null^ T cells. Other apoptosis inhibitors, such as *BIRC2* (encoding cIAP1 protein) and *BIRC3* (encoding cIAP2 protein), are upregulated while the caspase activator *DIABLO* is downregulated in CD28^null^ T cells. Thus, changes in the transcriptional regulation of apoptotic molecules and their regulators might be responsible for the resistance of CD28^null^ T cells to apoptosis. Modulating the fine balance between apoptotic signals might be a useful objective to avoid the accumulation of these cells over time. In fact, proteasome inhibitors are known to restore the sensitivity of CD28^null^ T cells to apoptosis, thereby favoring their elimination (Kovalcsik *et al*., 2015). Our results suggest that other forms of cell death, such as pyroptosis or necroptosis, could also be misrepresented in these cells, although this requires further detailed investigation.

In summary, we demonstrate that loss of the CD28 molecule in CD4^+^ T lymphocytes during aging is associated with profound DNA methylation changes that contribute to the acquisition of the unique biological and functional properties observed in this cell subset. Differentially methylated regions are strongly associated with gene expression changes, leading to changes in the cytotoxic response, TCR signaling, and cell death pathways. Identifying the mechanisms regulating these pathways will provide a better understanding of how these pathogenic and ‘senescent’ CD28^null^ T cells are generated and suggest new strategies for blocking the function and longevity of these cells in the course of T‐cell aging.

## Experimental procedures

### Samples, cell separation, and reagents

Blood from healthy donors at different ages was obtained from the Asturias Transfusion Centre and the Internal Medicine Department of the Hospital Central de Asturias, Spain, after obtaining informed consent in accordance with the Declaration of Helsinki and following approval by the hospital's Ethics Committee. Healthy status was defined as the absence of autoimmune diseases, cancer, infectious diseases, dementia, and cardiovascular diseases that could interfere with the study. Peripheral blood mononuclear cells (PBMCs) were isolated by density gradient centrifugation. CD28^null^ and CD28^+^ T cells were purified using a CD4 Multisort kit and CD28 MicroBead kit (Miltenyi Biotec, Bergisch Gladbach, Germany) or sorted with a BD FACSARIA II cytometer (BD Bioscience, San José, CA, USA) after staining with CD4‐APC and CD28‐FITC monoclonal antibodies (Abs; BioLegend, San Diego, CA, USA). Purity was > 95% for all samples. Staurosporine (50 nm; Enzo Life Science, Lausen, Switzerland) was added to isolated PBMCs for 18 h to induce apoptosis.

### DNA and RNA extraction

Genomic DNA and total RNA were isolated from samples using the QiAmp DNA Micro kit (Qiagen, Hilden, Germany) and RNAqueous^®^ Micro kit (Thermo Fisher Scientific, Waltham, MA, USA), respectively, following the manufacturers’ instructions. Quality and quantity were assessed using an Agilent 2100 Bioanalyzer (Agilent Technologies, Santa Clara, CA, USA) and a fluorometric Qubit^®^ 2.0 assay (Invitrogen, Carlsbad,CA,USA), respectively.

### Whole‐genome DNA methylation arrays

Samples were pooled using the same DNA quantity for each donor. DNA methylation was analyzed using an Illumina Infinium HumanMethylation27 BeadChip array (Illumina Inc. San Diego, CA, USA). Genomic DNA (500 ng) was bisulfite‐converted with the EZ DNA methylation kit (Zymo Research Corp. Irvine, CA, USA) following the manufacturer's protocol, then whole‐genome amplified, enzymatically fragmented, and purified to hybridize to the locus‐specific oligonucleotides on the BeadArray. Fluorescent signals were measured with the BeadStation GX scanner (Illumina Inc.), and raw data were decoded with GenomeStudio software (Illumina Inc.). The amount of methylated DNA for a specific cytosine is represented by the average beta value, which ranges between 0 (completely unmethylated) and 1 (completely methylated). Datasets were generated from two biological replicates per cell type (CD28^null^ and CD28^+^), each obtained from a pool of 12 healthy donors.

### Whole‐genome gene expression arrays

Samples were pooled using the same RNA quantity for each donor, and their gene expression levels were characterized using Human HT12 v3 BeadChips (Illumina Inc.). cRNA was synthesized with a TargetAmpTM Nano‐gTM Biotin‐aRNA Labeling Kit for the Illumina R System (Epicentre, Illumina Inc. Madison, WI, USA), and subsequent amplification, labeling, and hybridization were performed following Illumina's protocol. Raw data were decoded with GenomeStudio software (Illumina Inc.) to obtain a final report (sample probe profile). Datasets were generated using the same pools of healthy donors as for the methylation arrays.

### Statistical and functional analysis

Raw methylation and expression data were background‐corrected, log_2_‐transformed, and quantile‐normalized using the r packages from the Bioconductor project and lumi package (Gentleman *et al*., 2004; Du *et al*., 2008). Probabilities were corrected for multiple testing by determining the false discovery rates (FDRs) with the Benjamin–Hochberg procedure. Probes with detection *P*‐values > 0.01 and sex chromosomes were excluded from the analysis. For methylation arrays, we used β values as a quantitative measure of DNA methylation levels of specific CpGs. To analyze the differentially methylated regions (DMRs), we considered probes with values of FDR‐adjusted *P* < 0.01 and β ≥ 0.7 (70%, hypermethylated probes) or ≤ 0.3 (30%, unmethylated probes). To compare subsets, we only considered probes in CD28^null^ T cells showing a difference of ≥ 20% (Δβ ≥ 0.2) relative to their CD28^+^ counterpart. For expression data, a gene was considered differentially expressed if it had a value of FDR‐adjusted *P* < 0.05 and a > 1.5‐fold change (FC) in expression. Gene ontology (GO) enrichment was performed with the web‐based DAVID GO tool (Huang da *et al*., 2009). Raw array data have been submitted to the NCBI Gene Expression Omnibus (GEO) under accession number GSE78942 (https://www.ncbi.nlm.nih.gov/geo/query/acc.cgi?acc=GSE78942).

### Bisulfite pyrosequencing

Genomic DNA (500 ng) was bisulfite‐converted with an EZ DNA methylation kit (Zymo Research Corp.) following the manufacturer's protocol. Primers were designed with pyromark assay design 2.0 software (available upon request). Pyrosequencing was performed with the pyromark kit and the Pyrosequencing Vacuum Prep Tool (Qiagen). Methylation was quantified using the pyromark Q24 system (Biotage, Uppsala, Sweden).

### Quantitative real‐time PCR

Total RNA was transcribed with the High Capacity cDNA Reverse Transcription kit (Applied Biosystem). Gene expression was quantified by real‐time PCR on a Step One Plus RT–PCR System (Applied Biosystems) using TaqMan assays (Applied Biosystems, Foster City, CA, USA) or designed primers (Table S5, Supporting information). The numbers of transcripts were calculated using threshold cycle (Ct) values standardized to *GAPDH*, using the 2‐(ΔCt) method.

### TCR landscape

The global TCR repertoire was analyzed by TcLandscape, which combines the analysis of Vβ chain gene segment usage, CDR3 length distribution (*CDR3‐LD*) level and a quantitative PCR assessment of all *CDR3 length‐restricted* mRNAs in each family. For this purpose, mRNA from CD28^+^ and CD28^null^ T cells was isolated from two healthy donors. The analysis was carried out by TcLand Expression Inc., Huningue, France (www.tcland-expression.com). The results were displayed as a three‐dimensional TcLandscape^®^.

### Flow cytometry

The following Abs were used: CD4‐APC, CD3‐PERCP, CD28‐FITC, CX3CR1‐PE, CD27‐PE, Granzyme‐B‐PE, and isotype‐matched controls (BioLegend). For intracellular staining, cells were fixed, permeabilized with a Fixation/Permeabilization kit (Immunostep, Salamanca, Spain), and stained with anti‐Lck and antiphospho‐Zap‐70 Abs (Cell Signaling Technology, Danvers, MA, USA) and a secondary antibody conjugated to phycoerythrin (Biolegend). The active form of caspase‐1 was determined with the FAM‐FLICA *in vitro* caspase‐1 detection kit (ImmunoChemistry Technologies, Bloomington, MN, USA). Apoptosis was quantified by staining with CD28‐PE and CD4‐APC and FITC Annexin V/7AAD Apoptosis detection kit, following the manufacturer's instructions. Cells were analyzed on a Gallios Flow Cytometer (Beckman Coulter, Inc. Fullerton, CA, USA)

### Inflammasome activation and IL‐1β ELISA

To activate NLRP3 inflammasome, cells (2 × 10^5^) were primed with TNF‐α (5 ng mL^−1^; PeproTech EC Ltd. London, UK) for 1 h at 37 °C, followed by treatment with nigericin (10 μm; InvivoGen, San Diego, CA, USA) overnight. Free‐cell culture supernatants were collected, and human IL‐1β (Biolegend) levels were quantified by ELISA, following the manufacturer's instructions.

## Funding

This work is supported by Plan Nacional de I+D+I 2008–2011 and European Union Fondos Feder; Instituto de Salud Carlos III (grant number PI12*/*02587 and PI16/01318); Red Española de Investigación Renal (REDinREN) (grant number RD12*/*0021*/*0018 and 0021, and RD16/0009/0020), Plan de Ciencia, Tecnología e Innovación 2013‐2017 del Principado de Asturias (reference GRUPIN‐14‐030), by the Government of the Basque Country (Etortek Research Programs 2008/2015) the Innovation Technology Department of Bizkaia.

## Author contributions

BSA and CLL designed the experiments and wrote the manuscript; BSA, RMR, KS, ABR, and LM performed the experiments and discussed the results; CDC provided the samples; and AF and AMA helped analyze the data.

## Conflict of interest

The authors have no conflicts of interests.

## Supporting information


**Fig. S1** Gene expression changes between CD28^+^ and CD28^null^ T cells and functional analysis.
**Fig. S2** Gene ontology analysis of genes upregulated and downregulated in CD28^null^ T cells.
**Fig. S3** Apoptosis analysis in CD28^null^ T cells.
**Fig. S4** Reproducibility of the methylation profiles in the biological replicates of CD28^+^ and CD28^null^ T cells subsets.
**Fig. S5** Biological process enrichment in CD28^null^ T cells taking differentially methylated genes into account.
**Fig. S6** Association of the DNA methylation and gene expression changes in CD28^null^ T cells.
**Fig. S7** Defects in the Lck and phosphorylated ZAP‐70 expression in CD28^null^ T cells.

**Table S1** Genes differentially expressed (DEGs) in CD28^null^ T cells.

**Table S2** Gene ontology analysis of genes differentially expressed genes in CD28^null^ T cells.

**Table S3** Regions differentially methylated between CD28^+^ and CD28^null^ T cells.

**Table S4** Gene ontology analysis of regions differentially methylated in CD28^+^ and CD28^null^ T cells.

**Table S5** RT‐PCR TaqMan assays and primers.
