# Supplementary material for: Phenotypic characteristics of aged CD4+ CD28null T lymphocytes are determined by changes in the whole‐genome DNA methylation pattern
Source: Aging Cell. 2016 Dec 27;16(2):293–303. doi: 10.1111/acel.12552 (PMC5334526; doi:10.1111/acel.12552)
Supplement: Supplementary file 1 — Fig. S1 Gene expression changes between CD28+ and CD28null T cells and functional analysis. Fig. S2 Gene ontology analysis of genes upregulated and downregulated in CD28null T cells. Fig. S3 Apoptosis analysis in CD28null T cells. Fig. S4 Reproducibility of the methylation profiles in the biological replicates of CD28+ and CD28null T cells subsets. Fig. S5 Biological process enrichment in CD28null T cells taking differentially methylated genes into account. Fig. S6 Association of the DNA methylation and gene expression changes in CD28null T cells. Fig. S7 Defects in the Lck and phosphorylated ZAP‐70 expression in CD28null T cells. [file ACEL-16-293-s001.pdf]

Figure S1

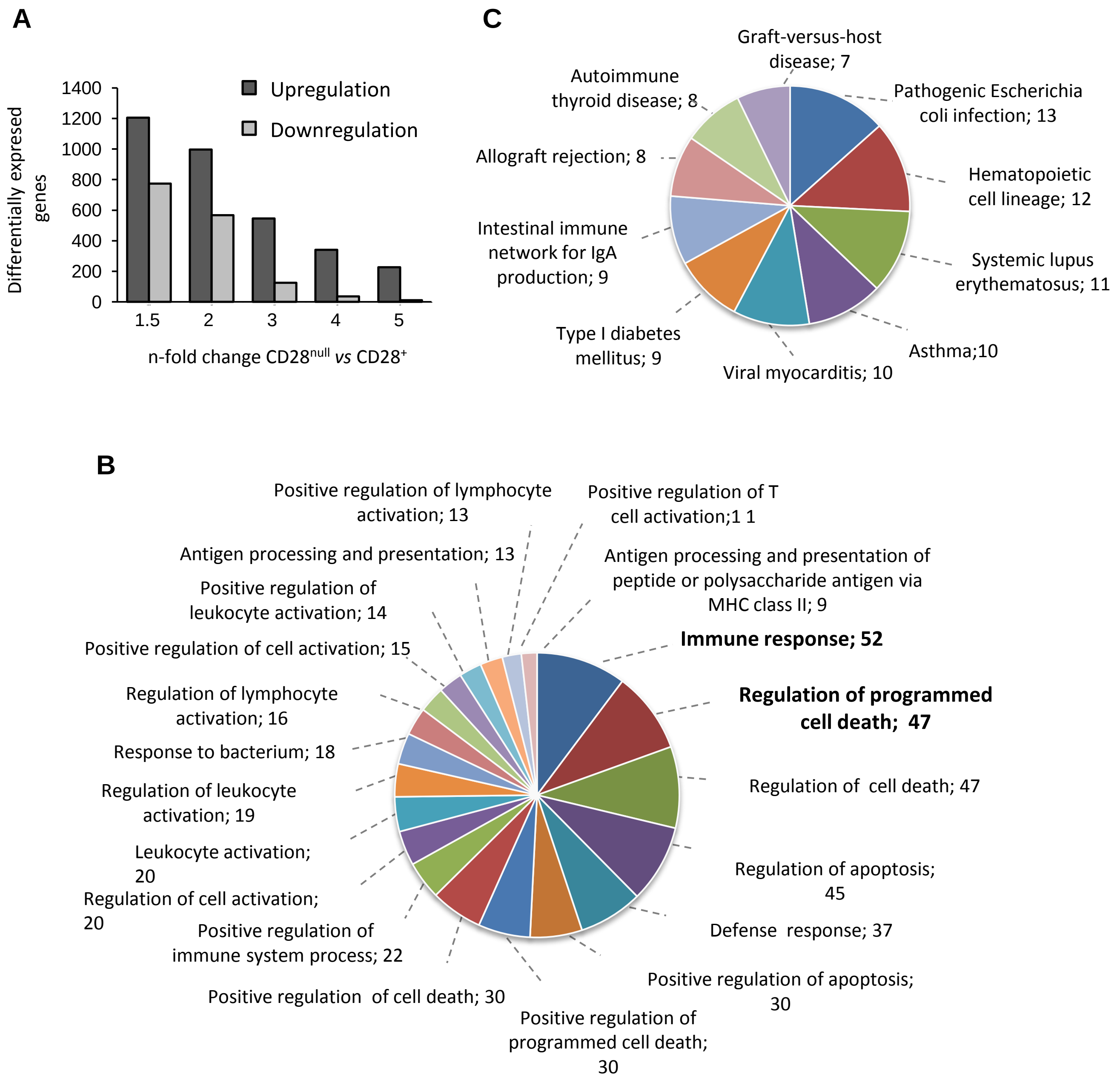

Figure S1.- Gene expression changes between CD28$^+$ and CD28^{null} T cells and functional analysis. A) The distribution of differentially expressed genes (DEGs) between the two CD4+ T cell subsets (CD28^{null} *vs.* CD28$^+$). Histograms represent the number of genes in each n-fold change size with adjusted $p < 0.01$. **B)** Gene ontology (GO) analysis of all DEGs (545 upregulated and 125 downregulated genes) in CD28^{null} T cells with adjusted $p < 0.01$ and FC ≥ 3 or ≤ -3. The 20 most significant categories are shown ($p \leq 2.2 \times 10^{-4}$) and the number of genes in each category is indicated. **C)** KEGG analysis of all DEGs with $p < 0.01$. The 10 most significant categories and gene numbers in each are shown.

Figure S2

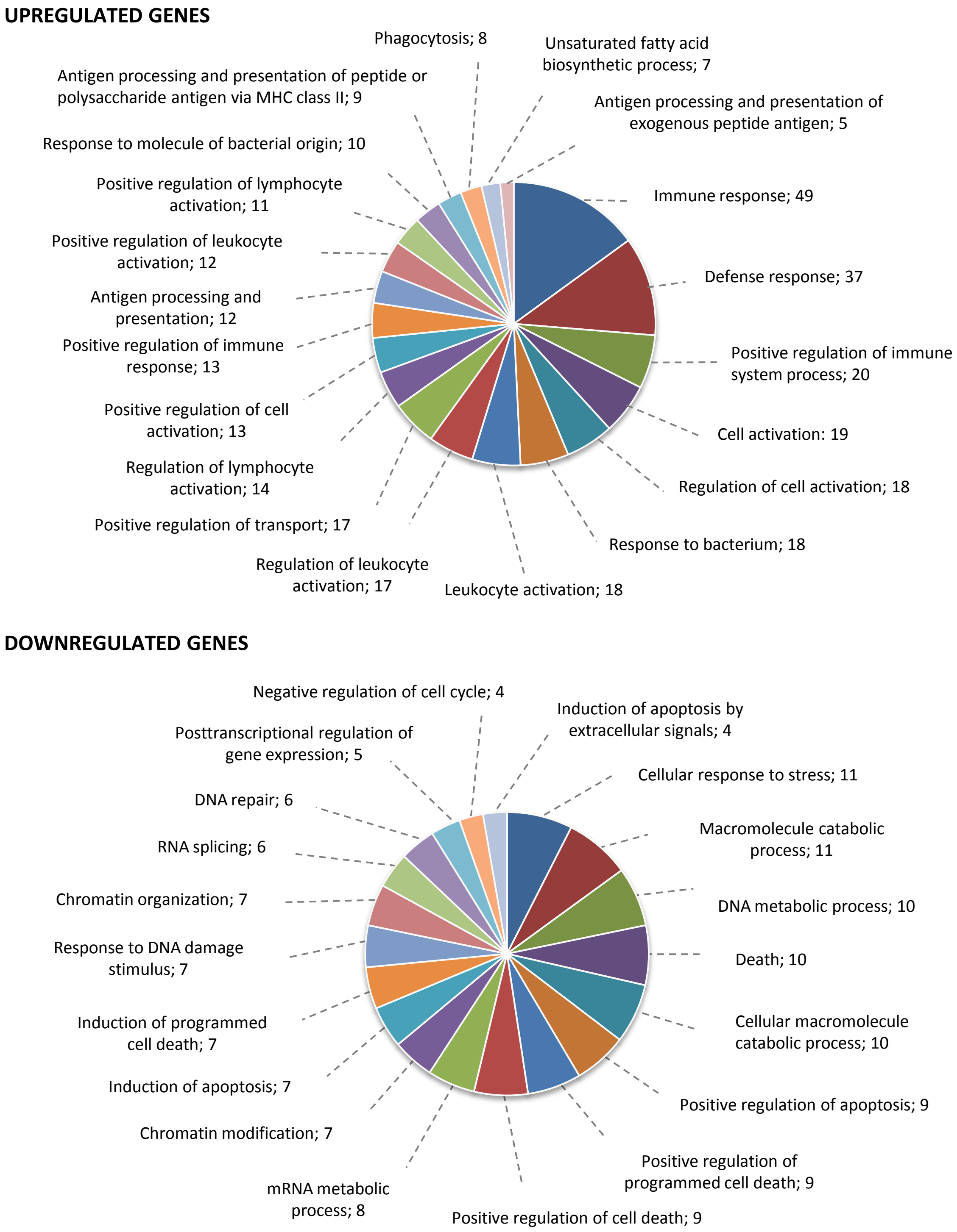

Figure S2.- Gene Ontology analysis of genes upregulated and downregulated in CD28[null] T cells. Pie charts showing the most enriched biological processes of the 545 upregulated and 125 downregulated genes in CD28[null] T cells using the criteria of adjusted $p < 0.01$ and FC ≥ 3 or ≤ -3. The number of genes in each category is indicated. Upregulated genes are mainly enriched in biological pathways associated with the immune and defense responses (GO: 0006955 and GO: 0006952). Downregulated genes show a wider range of functions.

Figure S3

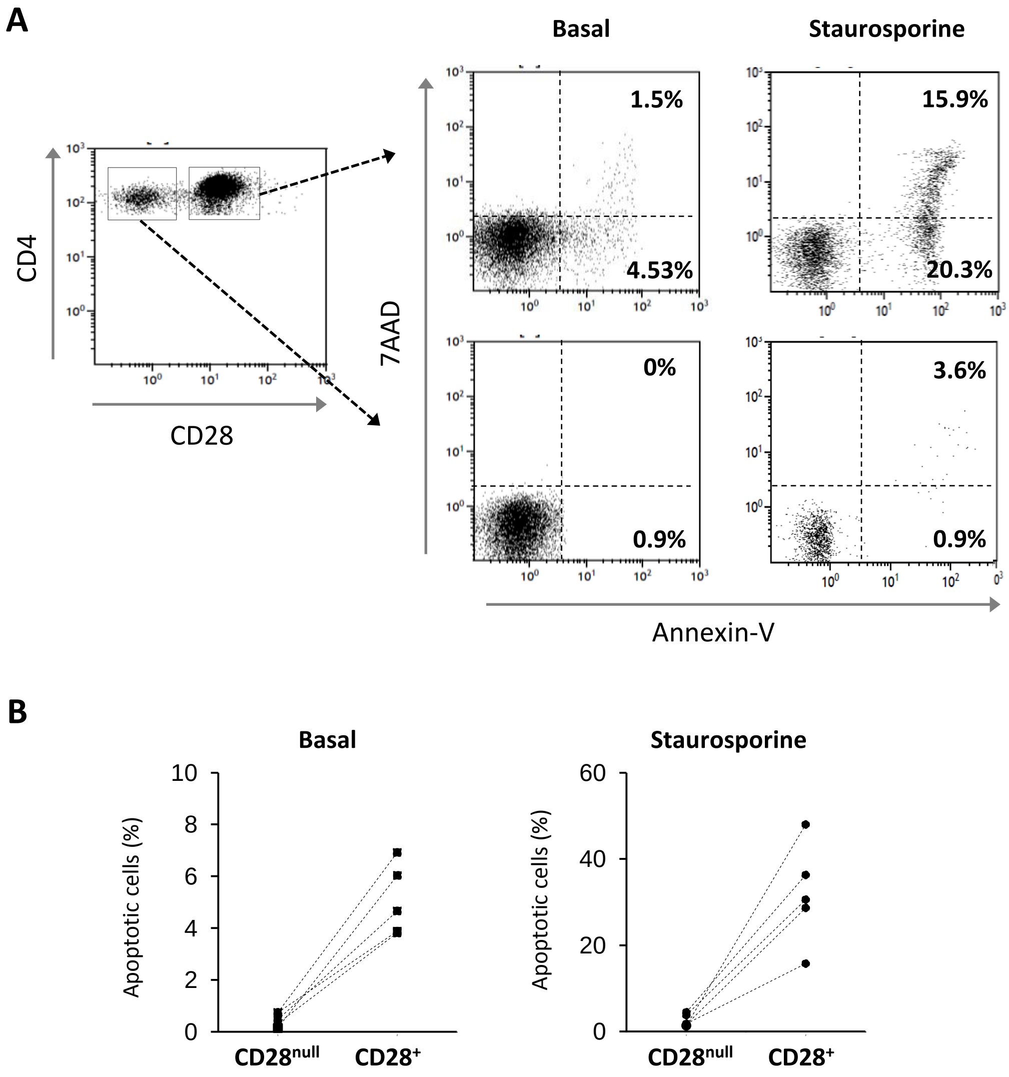

Figure S3.- Apoptosis analysis in CD28null T cells. PBMCs isolated from healthy donors were culture without or with staurosporine (50nm) for 18h and apoptosis was quantified by the percentage of cells stained with Annexin-V+ / 7AAD+ (late apoptosis) and Annexin-V+ /7AAD- (early apoptosis) within the CD28^{null} and CD28^{+} T cell subsets. **A)** Representative dot plots for apoptosis analysis by flow cytometry. Numbers indicates the percentage of cells in each quadrant. B) Percentage of apoptotic cells in CD28^{+}; and CD28^{null} T cell subsets from 5 healthy donors.

Figure S4

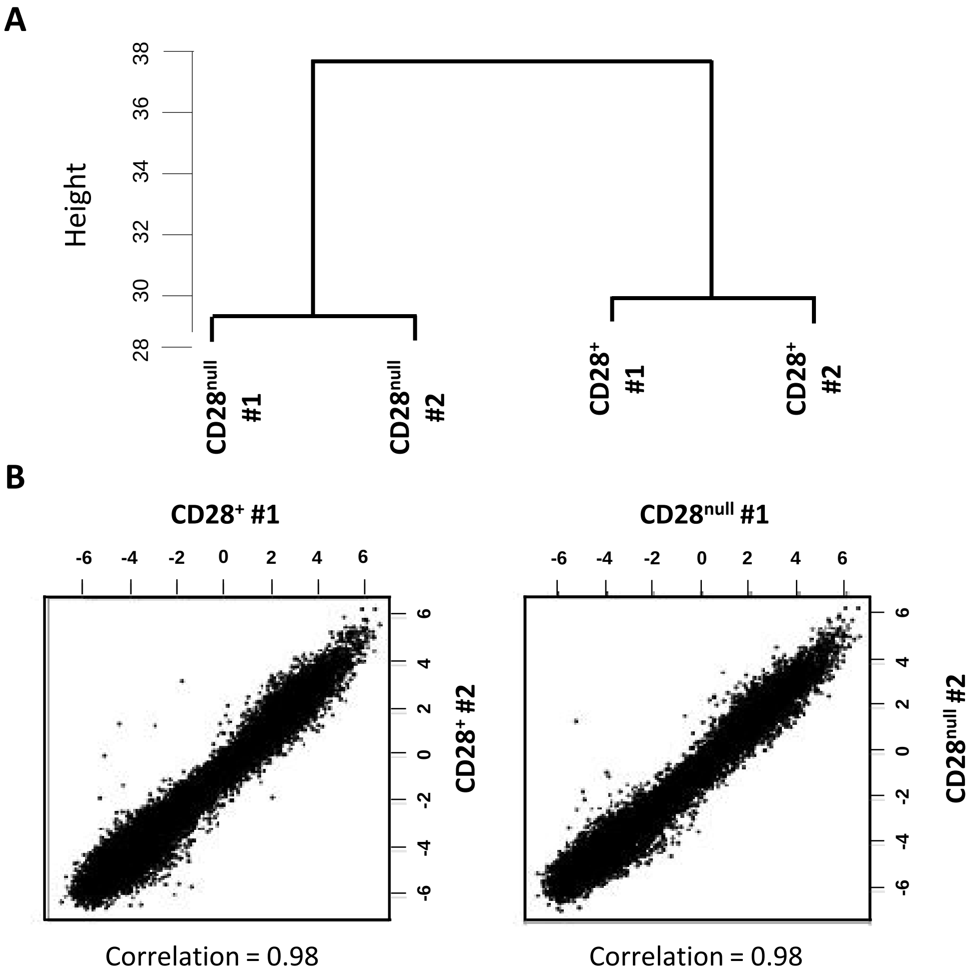

Figure S4.- Reproducibility of the methylation profiles in the biological replicates of $CD28^{+}$ and $CD28^{null}$ T cells subsets. Cell subsets were isolated from 24 healthy donors and grouped into two identical pools (12 individuals each). Samples were pooled using the same DNA quantity per donor. Unsupervised clustering (A) and scatterplot analysis (B) show the reproducibility of the two pools in both cell types using the Illumina methylation array.

Figure S5

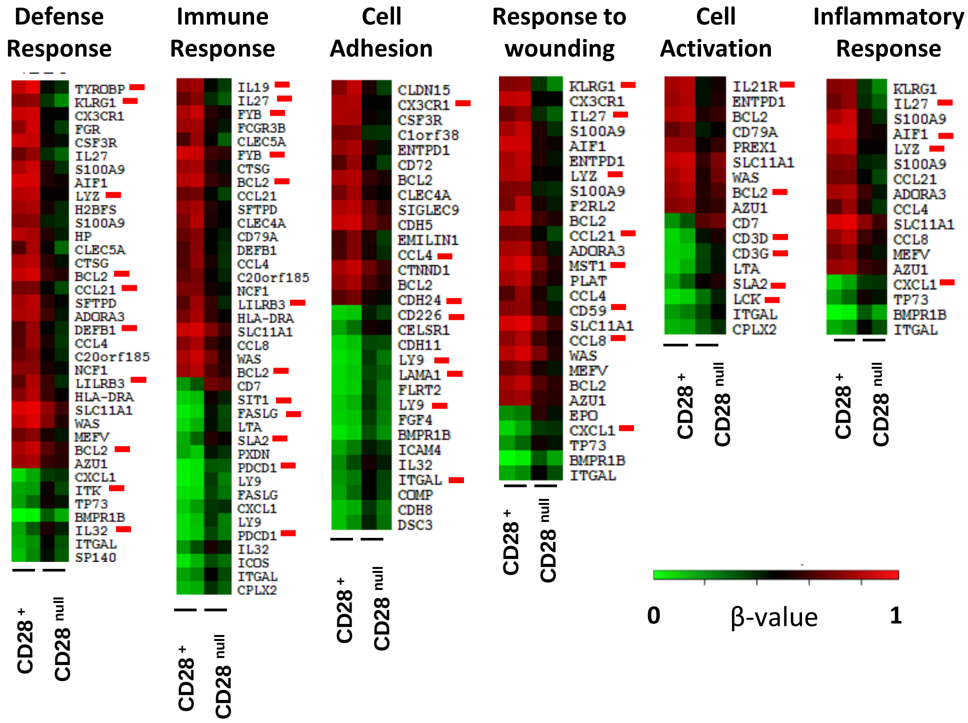

Figure S5.- Biological process enrichment in CD28null T cells taking differentially methylated genes into account. Heat maps of the functional categories altered in CD28null T cells: defense response (GO: 0006952), immune response (GO: 0006955), response to wounding (GO: 0009611), cell adhesion (GO: 0007155), cell activation (GO: 0001775) and inflammatory response (GO: 0006954). GO analysis was performed with the 160 demethylated genes and 136 *de novo*-methylated genes) with an adjusted $p < 0.01$. Representative genes of each category are marked in red. Data were obtained from the two biological replicates for each cell type. Methylation levels vary from unmethylated ($\beta = 0$, red) to fully methylated ($\beta = 1$, green).

Figure S6

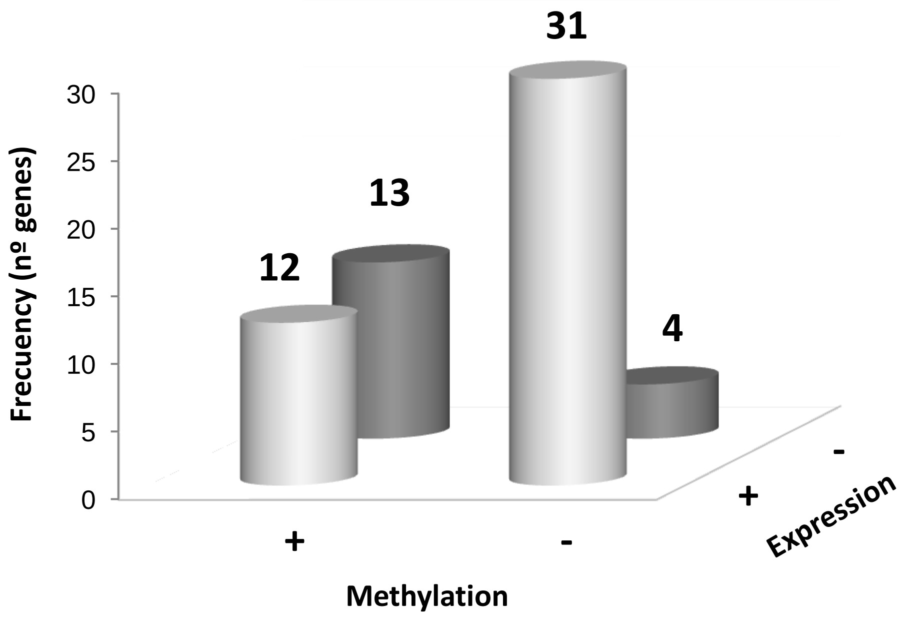

Figure S6.- Association of the DNA methylation and gene expression changes in CD28^{null} T cells. Correlation between the differentially methylated regions and expression of their associated genes. Data from 170 genes were obtained from the Illumina methylation and expression arrays. Each bar shows the number of differentially methylated genes associated with changes in their gene expression (adjusted $p < 0.01$ and FC ≥ 1.5 or ≤ -1.5). (+) indicates *de novo* methylation or upregulated gene expression. (−) represents demethylation or downregulated gene expression.

Figure S7

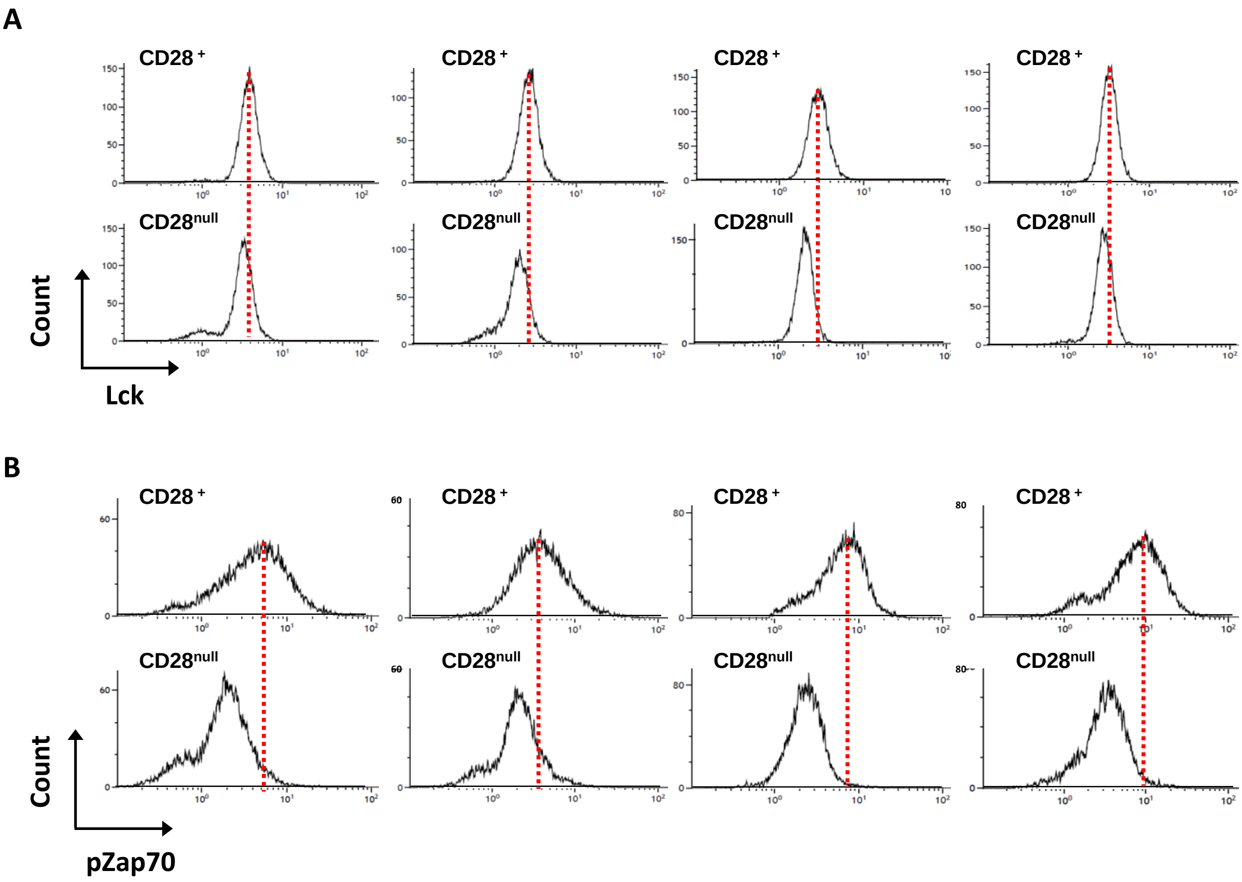

Figure S7.- Defects in the Lck and phosphorylated ZAP-70 expression in CD28^{null} T cells. Lck protein tyrosine kinase expression (**A**) and phosphorylation of Zap70 (pZap-70) (**B**) was analyzed by flow cytometry in four healthy donors. Dotted vertical lines indicate the differential expression between CD28+ and CD28null T cells for each individual.
