# Supplementary material for: Phenotypic characteristics of aged CD4+ CD28null T lymphocytes are determined by changes in the whole‐genome DNA methylation pattern
Source: Aging Cell. 2016 Dec 27;16(2):293–303. doi: 10.1111/acel.12552 (PMC5334526; doi:10.1111/acel.12552)
Supplement: Supplementary file 6 — Table S5 RT‐PCR TaqMan assays and primers. [file ACEL-16-293-s006.doc]

| **Gene** | **Taqman assay ID** |  |
| --- | --- | --- |
| *IL1B* | Hs01555410_m1(FAM) |  |
| *IL18* | Hs01038788_m1(FAM) |  |
| *CASP-1* | Hs00354836_m1(FAM) |  |
| *CARD8* | Hs01088221_m1(FAM) |  |
| *NLRP3* | Hs00918082_m1(FAM) |  |
| *PYCARD* | Hs01547324_gH(FAM) |  |
| *GAPDH* | Hs03929097_s1 (VIC) |  |
|  | **Forward** | **Reverse** |
| *TYROBP* | GCTGGCTGTAAGTGGTCTCC | TAAGGCGACTCGGTCTCAGT |
| *TXK* | GGAAGGCAAGAGACCGTTTG | TTATGGCAGCCTCCGTACTT |
| *ITK* | ACAGGAATGGGCATGAAGGA | GACACGGTGTATGTTCCTGC |
| *GAPDH* | TCGGAGTCAACGGATTTGGTCGT | TGCCATGGGTGGAATCATATTGGA |

Table S5.- **RT-PCR Taqman assays and primers**
